# Supplementary material for: Morphological and Phylogenetic Characterisations Reveal Four New Species in Leptosphaeriaceae (Pleosporales, Dothideomycetes)
Source: J Fungi (Basel). 2023 May 26;9(6):612. doi: 10.3390/jof9060612 (PMC10299561; doi:10.3390/jof9060612)
Supplement: Supplementary file 1 [file jof-09-00612-s001.zip › jof-2369606-supplementary.pdf]

Table S1. Synopsis of sexual and asexual morphological characteristics of *Leptosphaeria* species with molecular data.

Species name	Host	Country	Ascomata	Asci		Ascospore			References
Sexual morph			Size (μm)	Size (μm)	Shape	Size (μm)	Shape	Septa	
L. chatkalica	Unknown	Uzbekistan	306–400 × 380–500	130–190 × 9–12	Cylindrical, long with club-like pedicellate.	20–30 × 6–9	Fusiform with narrow to acute at both ends, initially hyaline to yellowish-brown at maturity, mucilaginous sheath.	3	[5]
L. cichorii	*Cichorium intybus*	Italy	206–240 × 251–363	71–115 × 5–8	Cylindrical, short pedicellate.	11–20 × 3–6	Fusiform with acute ends to narrow rounded ends, reddish to yellowish-brown.	3	[2]
L. cirsii	*Cirsium* sp.	Italy	285–315 × 335–360	75–110 × 10–12	Cylindric-clavate, short and broad pedicellate.	19–22 × 4–4.5	Ellipsoidal with narrow, rounded ends, initially hyaline, light brown to chestnut-brown at maturity, mucilaginous sheath.	3	[74]
L. conoidea	*Solidago* spp.	Europe, North America	Up to 500 in diameter	75–135 × 7–8	–	18–25 × 5–7	Ellipsoidal with obtuse rather than acute end cells, yellowish-brown.	3	[55]
L. doliolum	*Urtica dioica*	Italy, England	154–211 × 160–225	52–71 × 4–5	Narrowly cylindrical, short-bulbous pedicellate.	15–19 × 2–3	Fusiform with acute to narrow rounded ends, yellowish-brown to brown.	3	[2,18]
L. ebuli	*Sambucus embulus*	Italy	226–396 × 241–251	80–109 × 8–9	Clavate to sub-cylindrical, short and broad pedicel.	23–28 × 4–5	Ellipsoidal with broad rounded ends, initially hyaline, light brown to chestnut-brown at maturity.	3	[18]
L. errabunda	*Angelica* spp., *Solidago* spp.	Europe, North America	Up to 450 in diameter	80–100 × 6–8	Biseriate	20–35 × 4–4.5	Narrow, fusiform to ellipsoidal, brown, guttulate.	3	[55]

L. italica	*Rhamnus alpinus*	Italy	285–294 × 248–260	60–112 × 7–12	Cylindrical to cylindric-clavate, short pedicellate.	12–18 × 4–6	Ellipsoid to broadly fusiform, with rounded to acute ends, pale brown to yellowish-brown.	3	[75]
L. irregularis	Unknown	UK	245–370 × 180–368	110–147 × 6–8.5	Long-cylindric, short and furcate pedicellate.	23.5–29 × 5–7	Fusiform, conical at the ends, initially hyaline, reddish to yellowish-brown at maturity.	3	[74]
L. regiae	*Juglans regiae*	Turkey	315–377 × 364–410	99–130 × 9–10	Cylindrical to cylindric-clavate, short pedicellate.	15–18 × 6–7	Ellipsoid to broadly fusiform, with rounded to acute ends, initially hyaline, yellowish-brown to brown at maturity.	3	[41]
L. slovacica	*Ballota nigra*, *Leonurus cardiaca*	Netherlands	275–375 in diameter	60–100 × 5.5–7.5	Biseriate in the upper part, uniseriate below.	18–28 × 4.5–5.5	Fusiform with acute ends, olivaceous yellow.	3	[13,55]
L. sydowii	*Senecio* sp., *Papaver rhoeas*	Europe, UK, Switzerland, Netherlands	300–350 in diameter	80–90 × 11–12	–	24–30 × 6.5–7.5	Broadly fusiform, yellowish-brown with guttules.	3	[13]
L. urticae	*Urtica dioica*	UK	100–130 × 70–110	60–140 × 9–11	Cylindrical, pedicellate.	35–40 × 4–6	Long fusiform, initially hyaline, then yellowish-brown.	8–9	[3]
L. zhaotongensis	Herbaceous	China	380–550 × 185–300	84–118 × 7.7–11	Cylindrical to cylindric-clavate.	16.2–21.3 × 4.5–6.5	Fusiform, with narrow to acute at both ends, guttulate.	3	In this study
**Species name**	**Host**	**Country**	**Conidiomata**	**Conidiogenous cells**		**Conidia**			**References**
**Asexual morph**			**Size (µm)**	**Size (µm)**	**Shape**	**Size (µm)**	**Shape**	**Septa**	
L. cichorii	*Cichorium intybus*	Italy	189–200 × 196–220	2–5 × 2–4	Enteroblastic, phialidic, subglobose to short conical.	3–6 × 1–3	Hyaline, oblong to elliptic, thin-walled, smooth, guttulate.	0	[2]
L. cirsii	*Cirsium* sp.	Italy	150–200 × 195–220	2–5 × 2–4	Enteroblastic, phialidic, determinate integrated, subglobose to short conical.	3–6 × 1–3	Hyaline, oblong to cylindrical, thin-walled, smooth, guttulate, rounded at both ends.	0	[74]

L. conoidea	*Solidago* spp.	Europe, North America	250–800 × 150–300	–	–	5–7 ×2 –3 or 7–12 × 2-3	Ellipsoidal to subcylindrical, eguttulate or with 2 small polar guttules.	1	[55]
L. macrocapsa	*Mercurialis perennis*	Europe, Netherlands	400–600	–	–	3–5 × l–2	Ellipsoidal to subcylindrical, usually with two conspicuous polar guttules.	–	[13,55]
L. pedicularis	*Pedicularis* spp., *Gentiana* spp.	Europe, Switzerland	300–600 in diameter	–	–	4–8.5 × 2–3 or 4–6 × 2–4.5	Oblong to ellipsoidal, subcylindrical or allantoid, or oval ovoid or subglobose.	–	[13,55]
L. sclerotioides	*Melilotus albus*, *Medicago sativa*	Eurasia, North America, Canada	200–1000 in diameter	–	–	4.5–6 × 1.5–3	Ellipsoidal to subcylindrical, eguttulate or with 1–4 polar guttules.	–	[13,55]
L. slovacica	*Ballota nigra*, *Leonurus cardiaca*	Europe	200–300 in diameter	–	–	3.5–5.5 × 1.5–2.5	Oblong to ellipsoidal, with 2 minutes polar guttules.	–	[55]
L. sydowii	*Senecio* sp.	Europe, UK	300–450 in diameter	–	–	4–8.5 × 1.5–2.5	Irregular, oblong-ellipsoidal to subcylindrical, with 2-several small, more or less polar guttules.	–	[55]
L. veronicae	*Veronica* sp.	Netherlands	200–350 in diameter	–	–	3.5–6 × 1–2	Ellipsoidal to subcylindrical, with2-several more or less polar guttules.	–	[13,55]
L. yunnanensis	Herbaceous	China	280–515 diam × 170–370 high	2.8–6.4 × 3.8–6.7	Ampulliform or globose to subglobose,	2.5–6.6 × 1–1.8	Ellipsoidal to sub-cylindrical with obtuse ends.	0	In this study

Remarks: "–" means Not reported.

Table S2. Synopsis of sexual and asexual morphological characteristics of *Paraleptosphaeria* species with molecular data.

Species name	Host	Country	Ascomata	Asci		Ascospores			References
Sexual morph			Size (μm)	Size (μm)	Shape	Size (μm)	Shape	septa	
Pa. dryadis as *Leptosphaeria dryadophila*	*Dryas octopetala*	Switzerland	150–250 × 180–275	72–118 × 10.8–13.6	Cylindrical, thin-walled but with a thickened rounded apex, short-stalked.	20.1–24.5 × 5.7–7.2	Fusiform to slightly clavate. with acute end-cell shape, slightly curved.	3(–4–5)	[13,76]
Pa. kunmingensis	Herbaceous	China	215–300 × 145–220	70–104 × 12–16.4	Cylindrical to cylindric-clavate, short pedicellate with club-like pedicel, with ocular chamber	33.5–50.5 × 5–7.2	Lunate to long fusiform or inequilateral, straight or slightly curved.	1–3	In this study
Pa. macrospora	*Rumex domesticus*	Norway	-	105 × 18	Oblongish	44 × 8	Cylindrical-fusiform	3	[13, 54]
Pa. nitschkei	Petasites, *Cirsium spinosissimum, Cacalia* sp.	Italy, Switzerland, Austria	200–400	80–100 × 11–13	–	26–40 × 4.5–6	–	–	[13, 18,77]
Pa. padi	*Prunus padus*	Russia	288–345 × 330–396	74–127 × 8–14	Bitunicate, broad-cylindrical, with club-shaped pedicel, apically rounded, ocular chamber clearly visible when immature.	16–21 × 6–7	Broad-fusiform, conical at the apex, constricted at the septa, pale brown.	(2)–3	[19]
Pa. polylepidis	*Polylepis tarapacana*	Bolivia	300–700 × 300–600	120–250 × 20–45	Bitunicate, cylindrical-clavate, slightly curved, with a short stipe, apically widened, with an ocular chamber.	45–60 × 14–23	Pale brown, broadly ellipsoidal, narrower at the top, constricted at septa, uniformly thin-walled,	(2–)3 (–4)	[20,78]

							without perispore or gelatinous coat.		
Pa. praetermissa	*Rubus idaeus*	Sweden	–	110 × 10–11	Cylindrical	18–32 × 7–10	–	3	[13,54]
Pa. rubi	*Rubus* sp.	Italy	150–200 × 200–250	60–80 × 10–15	Bitunicate, fissitunicate, cylindrical, short pedicel, with an ocular chamber.	20–30 × 5–15	Hyaline, ellipsoidal, deeply constricted at the septum, straight or slightly curved, guttulate.	1	[2]
**Species name**	**Host**	**Country**	**Conidiomata**	**Conidiogenous cells**		**Conidia**			**References**
**Asexual morph**			**Size (μm)**	**Size (μm)**	**Shape**	**Size (μm)**	**Shape**	**septa**	
Pa. orobanches	*Epifagus virginiana*	USA	130–370 × 110–333	–	–	2–4 × 1–1.5	Ellipsoidal to oblong, sometimes guttulate.	–	[13,79]
Pa. rumicis	*Rumex alpinus*	France	up to 300	7–15 × 5–7	Hyaline, smooth, ampulliform.	32–70 × 2–2.5	Hyaline, granular, acicular, straight to gently curved, tapering towards apex that is acutely rounded.	up to 8	[17,80]

Remarks: "–" means Not reported.

Table S3. Synopsis of sexual and asexual morphological characteristics of *Plenodomus* species with molecular data.

**Species name**	**Host**	**Country**	**Ascomata**	**Asci**		**Ascospore**			**References**
**Sexual morph**			**Size (μm)**	**Size (μm)**	**Shape**	**Size (μm)**	**Shape**	**Septa**	
Pl. agnitus	*Eupatorium cannabinum*	Netherlands	350–500	110–125 × 9–11	–	31–35 × 4–5	Narrowly subcylindrical, third cell from above slightly swollen,	6	[13,55]

							yellowish brown with 2 guttules per cell.		
Pl. artemisiae	*Artemisia* sp.	China	140–280 × 300–450	64–100 × 9.5–13	Fissitunicate, cylindrical, short, pedicellate, apically rounded with a distinct ocular chamber.	28–40 × 4.5–6	Pale brown, fusiform, slightly constricted at the septa, enlarge at the third cell from above, lacking a mucilaginous sheath and appendages.	5	[3]
Pl. biglobosus	*Brassica juncea*	France	280–350	100–140 × 12–20	Bitunicate, cylindrical-clavate,	42–60 × 6–7	Fusiform.	3–5	[81]
Pl. collinsoniae	*Vitis coignetiae*	Japan	270-350	75 × 10	Cylindrical, short-stipitate.	30–32 × 4	Amber colored, straight or nearly so, widest in the middle, gradually narrowing to the subacute ends.	5–8	[13,54, 82]
Pl. confertus	*Anacyclus radiatus*	Spain	–	140–150 × 16	–	50–55 × 6	–	3–5	[13,54]
Pl. congestus	Erigeron canadensis	Spain	380-400	92–120 × 12–14	Cylindrical	24–32 × 7–8	–	3	[13,83]
Pl. enteroleucus	*Pyrus communis, Triticum aestivum, Catalpa bignonioides*	France, Germany, Netherlands	–	–	–	4–1.5	Ovoidal, oblongish	–	[13,84]
Pl. fallaciosus	Satureia montana	France	200-300	70-14	Oblongish, clavate	47–50 × 4–5	Cylindrical	7	[13,85]

Pl. guttulatus	Dead stem	Germany	230–325 × 260–310	70–72 × 5–8	Fissitunicate, cylindrical, with a bulbous pedicel, rounded at the apex, with an ocular chamber.	22– 24 × 2–3	Long fusiform, not or slightly constricted at each septum, cell above central septum slightly wider, guttulate only at the central cells, lacking a mucilaginous sheath.	5	[2]
Pl. hendersoniae	*Salix cinerea*, *Pyrus malus*	Sweden, Netherlands	–	96 × 12	Cylindrical	16 × 6	Oblongish	3	[13,86]
Pl. libanotidis	*Seseli libanotis*	Sweden	–		Elongated	20 × 8	Oblongish, fusiform.	3	[13,87]
Pl. pimpinellae	*Pimpinella anisum*	Israel	300–400 × 500	90–120 × 14–16	Clavate, short pedicellate	50–65 × 5.5–6.5	Fusiform	5–6(–10)	[13,88]
Pl. salviae	*Salvia glutinosa*	Italy	130–250 × 150– 280	57–88 × 8.6–11.4	Fissitunicate, cylindrical, with short bulbous pedicel, rounded at the apex, with a distinct ocular chamber.	30–48 × 3.1–4.3	Cylindric-fusiform, not or slightly constricted at each septum, lacking a mucilaginous sheath.	5	[2]
Pl. sinensis	fern, *Ageratina adenophora* , *Tamarindus indica*	China	250–290 × 300–360	75–107 × 8.5–12	Fissitunicate, cylindrical to cylindric-clavate, subsessile to short pedicellate, with knob-like to truncate pedicel.	30–39 × 4–6.5	Fusiform, widest at the third cell, slightly constricted at the septa, deeply constricted at the third septum from above, inconspicuous minute appendages at both end cells.	6	[3]

Pl. triseptatus	*Daucus carota* L.	Russia	276–324 × 217–250	58–112 × 7–13	Fssitunicate, cylindrical, short pedicel, with furcate ends, apically rounded, with welldeveloped ocular chamber.	19–25 × 5.7–7.2	Fusiform, with pointed ends, slightly constricted at the septa.	3	[40]
Pl. visci	*Viscum album*	France, Hungary	350	110–140 × 13–17	Cylindrical to subclavate, apically blunt rounded with a small ocular chamber.	28–38 × 6–8	Cylindrical to slightly clavate, upper part somewhat broader, lower part slightly tapered, end cells conical to rounded, olive.	(5–) 7	[13,39]
Pl. zhaotongensis	Herbaceous plant	China	200–300 × 210–320	80–132 × 10–14	Fissitunicate, bitunicate, cylindric-clavate, short pedicellate with foot-like pedicel, with ocular chamber, thick-walled at the apex.	34–42 × 3.5–6	lunate to long fusiform, straight or slightly curved,often constricted at medium septum, widest at the middle, rounded or slightly pointed at both ends.	6	In this study
**Species name**	**Host**	**Country**	**Conidiomata**	**Conidiogenous cells**		**Conidia**			**References**
**Asexual morph**				**Size (μm)**	**Shape**	**Size (μm)**	**Shape**	**septa**	
Pl. agnitus	*Eupatorium cannabinum*	Netherlands	125–250	–	–	4–7 × 1.5–2	Oblong-ellip-soidal, straight or slightly curved, with two inconspicuous guttules.	–	[13,55]
Pl. changchunensis	Poaceae sp	China	163–192 × 193–245	2.8–5.8 × 1.5–2.8	Enteroblastic, phialidic, determinate.	5-7.6 × 2-3.4	Oblong or oval, slightly curved toward the ends, rounded ends, hyaline, smooth-walled.	0	[6]

Pl. deqinensis	Soil	China	150–355 × 105–305	5–7 × 4–6.5	Phialidic, hyaline, smooth, ampulliform.	3.5–5.5 × 1.5–2.5	Ellipsoidal-cylindrical, smooth- and thin-walled.	0	[25]
Pl. hendersoniae	*Salix cinerea*, *Pyrus malus*	Sweden, Netherlands	–	–	–	16–18 × 6	–	3	[13,86]
Pl. influorescens	*Lilium* sp., *Fraxinus excelsior*	Netherlands	–	–	–	3–4 × 1–1.5	Ellipsoidal or ovoid.	–	[13,89]
Pl. lijiangensis	Fern	China	140–290 × 135–240	4–9 × 5–8	Enteroblastic, phialidic, discrete, determinate, ampulliform to doliiform, hyaline, smooth, with minute collarette, with 1–2 apertures.	3–5 × 1.7–2.3	Hyaline, oblong to obovoid, smooth-walled, with 1–2 guttules.	0	[3]
Pl. lupini	*Lupinus mutabilis*	Peru	–	–	–	2.5 × 1	Oblong	–	[13,90]
Pl. pimpinellae	*Pimpinella anisum*	Israel	300	–	–	4–4.5 × 1.2–1.8	Cylindrical	0	[13,88]
Pl. sinensis	Fern	China	50–120 × 50–110	3–8 × 4–8	Enteroblastic, phialidic, discrete, determinate, ampulliform to doliiform, collarette, and periclinal wall thickening, arising from the inner cavity of the conidioma wall.	2.7–4 × 1–2	Hyaline, oblong, slightly curved, smooth-walled.	0	[3]
Pl. tracheiphilus	*Citrus limonium*, *Citrus* sp.	Israel, Italy	100–180	–	Enteroblastic, phialidic, hyaline, simple, ampulliform, completely lining the inside of the pycnidial cavity.	2–3 × 1	Unicellular, straight or curved, with apices rounded.	0	[13,91]

Pl. visci	*Viscum album*	France, Hungary	100–150	–	–	4–6 × 2	Bacilliform, hyaline, guttulate, apex obtuse, base truncate.	0	[13,39]

Remarks: "–" means Not reported.
